# Profiling Ten RHO GTPases in Myeloid Malignancies Reveals Distinct Expression Patterns and Prognostic Associations

**DOI:** 10.1002/cam4.71770

**Published:** 2026-04-02

**Authors:** Beatriz de Almeida Rodrigues, Luciana Bueno de Paiva, Maria Carolina Clares Ramalho, Amanda Ferreira Damasceno, Sara Teresinha Olalla Saad, Mariana Lazarini

**Affiliations:** ^1^ Department of Clinical and Experimental Oncology Federal University of São Paulo (UNIFESP) São Paulo Brazil; ^2^ Hematology and Blood Transfusion Center, National Institute of Science and Technology of Blood (INCTS) University of Campinas (UNICAMP) Campinas Brazil

**Keywords:** acute myeloid leukemia, myelodysplastic neoplasms, RHO GTPases, RHOBTB2

## Abstract

The RHO GTPase family regulates cytoskeleton‐dependent processes, including proliferation and migration. Although their dysregulation is well described in solid tumors, little is known about their role in hematologic malignancies. We investigated the expression of ten RHO GTPase genes in bone marrow samples from patients with acute myeloid leukemia (AML) and myelodysplastic neoplasms (MDS) and analyzed TCGA AML data for prognostic associations. *RHOBTB2*, *RND2*, and *RHOQ* were differentially expressed compared with healthy controls. *RHOBTB2* was elevated in both MDS and AML and associated with inferior overall and disease‐free survival, including in intermediate‐risk AML. Our findings reveal distinct dysregulation patterns of RHO GTPases in myeloid malignancies and confirm *RHOBTB2* as a candidate prognostic marker in AML with a potential oncogenic role. These data support further investigation into the functional roles of RHO GTPases in leukemogenesis and their utility as emerging biomarkers in hematologic cancers.

## Graphical abstract

Expression of RHO GTPases in myeloid malignancies. Gene expression analysis of ten RHO GTPases revealed *RHOBTB2*, *RND2*, and *RHOQ* as differently expressed in patients with MDS and/or AML. *RHOBTB2* was identified as a promising prognostic indicator. BM, bone marrow. MDS, myelodysplastic neoplasm. AML, acute myeloid leukemia.

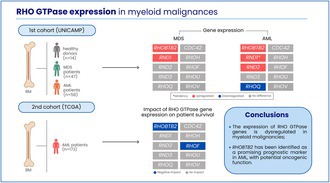

Acute myeloid leukemia (AML) is a severe hematological malignancy characterized by impaired blood cell production due to the abnormal growth of immature hematopoietic cells (myeloid blasts) in the bone marrow. AML typically affects adults, with a median age of diagnosis of 69 years [1]. The estimated 5‐year overall survival (OS) rate can range from 40% to 50% in young patients, while patients over the age of 60 show an average 5‐year OS rate of 20% to 30% [2].

Most AML cases occur without predisposing conditions (*de novo*). However, AML can also arise secondary to prior chemotherapy for unrelated malignancies or as a progression from other hematologic neoplasms [3]. Secondary AML cases are associated with worse clinical outcomes compared to *de novo* AML [4].

Myelodysplastic neoplasms (myelodysplastic syndromes or MDS) comprise a group of hematological disorders with cytological dysplasia in the bone marrow and a risk of evolution to AML [5]. Approximately 20% of newly diagnosed AML cases progress from MDS (AML with myelodysplasia‐related changes or AML‐MRC) [6]. Poor prognosis of AML‐MRC is related to escape of apoptosis, elevated percentages of myeloblasts in the bone marrow and specific genetic abnormalities [7, 8].

New treatment options for AML have been approved since 2017 for patients ineligible for standard chemotherapy [3]. However, approximately 10% to 40% of newly diagnosed patients with AML do not achieve complete remission with intensive induction therapy, and most patients relapse [9, 10]. In addition, the current therapy for MDS is not curative [11]. Therefore, the continued search for understanding molecular mechanisms underlying both diseases is imperative.

The human RAS superfamily, also known as small GTPases or G‐proteins, consists of over 150 members classified into five families, including the RHO family. Twenty human genes encode for RHO GTPases, which interact with approximately 1% of protein‐coding regions in the genome [12]. These proteins are grouped into subfamilies (RAC, RHO, CDC42, RHOD/RHOF, RHOBTB, RHOH, RHOU/V, and RND) based on their sequence homology [13].

RHO GTPases are well known as primary regulators of cytoskeletal dynamics, which in turn influence a variety of cellular activities related to leukemia development [14, 15]. The majority of RHO GTPases, categorized as “typical RHO GTPases,” are molecular switches that undergo a regulatory cycle consisting of binding to GTP (active state) or GDP (inactive state) [16]. In contrast, the “atypical GTPases” are predominantly bound to GTP. Alternative mechanisms, such as subcellular localization, post‐translational modifications, and gene expression, further regulate typical and atypical GTPases [17].

Dysregulation of RHO GTPases is often described across tumors and associated with patient prognosis [15, 18, 19]. Nonetheless, there is a significant knowledge gap concerning the expression of these genes in hematological neoplasms. In this study, we report the gene expression of 10 RHO GTPases and their impact on the survival of MDS, AML‐MRC, and *de novo* AML patients.

Bone marrow aspirates were obtained from the iliac crest of untreated patients with MDS with < 5% blasts (MDS‐LB, *n* = 31), MDS with > 5% blasts (MDS‐IB, *n* = 16), AML‐MRC (*n* = 15), and *de novo* AML (*n* = 43) at the Hematology and Blood Transfusion Center, University of Campinas, Brazil. Control samples were obtained from hematopoietic stem cell transplant donors (*n* = 14). MDS patients were classified according to the WHO 5th edition [20] and the revised international prognostic staging system (R‐IPSS) [21]. Clinical characteristics are in Table S1.

Mononuclear cells were isolated using Ficoll–Hypaque (Cytiva), followed by red blood cell lysis. Total RNA was extracted using TRIzol reagent, treated with DNase (Thermo Fisher Scientific), and reverse‐transcribed using the RevertAid First Strand cDNA Synthesis Kit (Thermo Fisher Scientific). All RNA samples were obtained from an institutional biorepository.

Gene expression was quantified by real‐time PCR using TaqMan probes (Table S2) and TaqMan Universal PCR Master Mix (Thermo Fisher Scientific), with *HPRT* as the endogenous control. Amplification was carried out on an ABI 7500 Real‐Time PCR System (Thermo Fisher Scientific). Relative expression was calculated using the 2^−ΔΔCT^ method [22], normalized to a healthy donor calibrator, and analyzed using the two‐tailed Mann–Whitney test (*p* < 0.05). All samples were included in the analyses, which were conducted using GraphPad Prism 10.0 (GraphPad Software).

We compared *RHOBTB2*, *RND1*, *RND2*, *RND3*, *RHOQ*, *CDC42*, *RHOH*, *RHOF*, *RHOU*, and *RHOV* expression between healthy donors and patients. Four of the ten genes presented significant differences in one or more groups (Figure 1).

**FIGURE 1:**
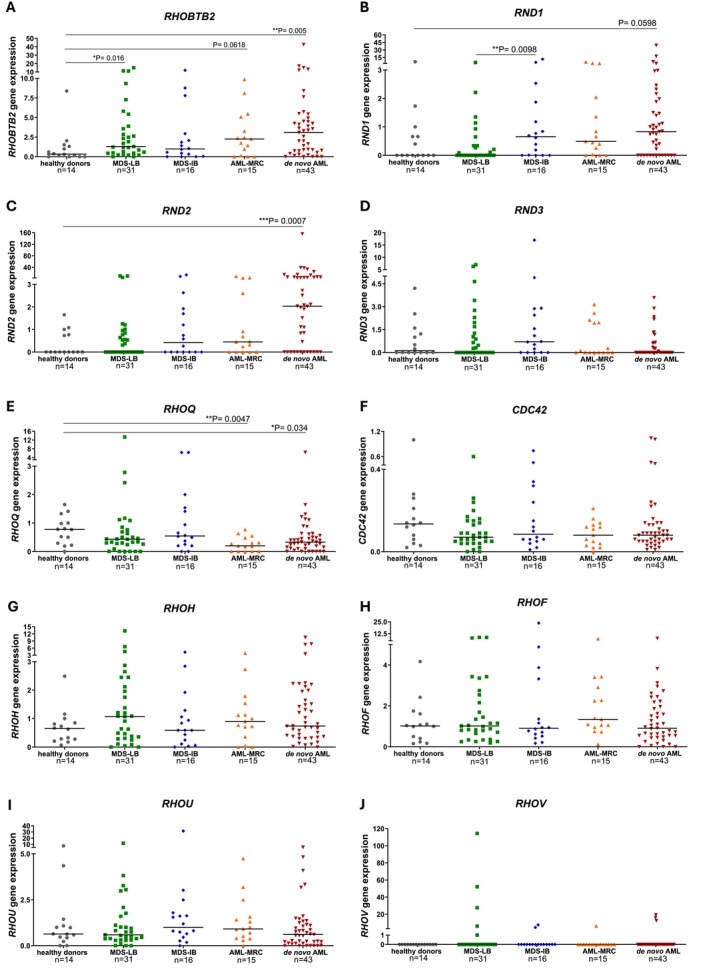
RHO GTPase expression in healthy donors and MDS, AML‐MRC, and *de novo* AML patients. (A) *RHOBTB2* expression was higher in MDS‐LB and in the *de novo* AML group in comparison to healthy donors. *RHOBTB2* also tended to be higher in AML‐MRC patients. (B) *RND1* expression was augmented in the MDS‐IB group in comparison with the MDS‐LB group. *RND1* tended to be higher in *de novo* AML when compared with healthy donors. (C) *RND2* gene expression was increased in *de novo* AML patients. (E) *RHOQ* expression was decreased in AML‐MRC and in *de novo* AML, in comparison with healthy donors. (D, F–J) *RND3*, *CDC42*, *RHOH*, *RHOF*, *RHOU*, and *RHOV* expressions did not significantly differ among the groups. Gene expression was evaluated by quantitative PCR in bone marrow samples. Each dot represents one subject and horizontal lines indicate medians. *HPRT* was used as a housekeeping gene. A sample from a healthy donor was used for calibration (value = 1). The numbers of subjects in each group and the *p* values (two‐tailed Mann–Whitney test) are indicated.

*RHOBTB2* expression was increased in *de novo* AML (median [range]: 3.10 [0.00–42.59], *p* < 0.01) and MDS‐LB group (0.99 [0.00–12.01], *p* < 0.05) compared with healthy donors (1.30 [0.00–15.08]). There was a trend toward elevated *RHOBTB2* in AML‐MRC (2.26 [0.00–9.86], *p* = 0.06) compared to healthy donors (Figure 1A).

*RND* subfamily expression was not detected in some bone marrow samples (Figure 1B–D). *RND1* expression was higher in MDS‐IB (0.66 [0.00–11.53], *p* < 0.01) compared with MDS‐LB (0.00 [0.00–5.10]) and tended to increase in *de novo* AML (0.83 [0.00–39.24], *p* = 0.06) compared with healthy donors (0.00 [0.00–6.50]) (Figure 1B). *RND2* was elevated in *de novo* AML (2.03 [0.00–155.80], *p* < 0.001) compared with healthy donors (0.00 [0.00–1.65]) (Figure 1C). *RHOQ* expression was reduced in AML‐MRC (0.20 [0.00–0.77], *p* < 0.01) and *de novo* AML (0.33 [0.00–6.33], *p* < 0.05) compared with healthy donors (0.77 [0.00–1.65]) (Figure 1E).

*RND3*, *CDC42*, *RHOH*, *RHOF*, and *RHOU* did not differ significantly among groups (Figure 1D,F–I). *RHOV* was undetectable in normal bone marrow and most patient samples (Figure 1J).

Spearman correlation revealed a moderate positive association between *RND1* expression and blast percentage in MDS (*r* = 0.4134, *p* = 0.039) and between *RND2* expression and blast percentage in AML (*r* = 0.4138, *p* = 0.0014). *RHOU* showed a weak negative correlation with blast percentage in AML (*r* = −0.3159, *p* = 0.0167). No genes showed strong or moderate correlation with patient age (data not shown).

We used a larger cohort from TCGA [23] to assess the impact of RHO GTPase expression on overall (OS) and disease‐free survival (DFS). Molecular profiling and clinical information were obtained from cBioPortal (RNA‐Seq V2 RSEM) (cbioportal.org/) [24, 25]. Patients were stratified by cytogenetic risk into favorable (*n* = 32), intermediate (*n* = 101), and adverse (*n* = 37). For DFS, 121 of 173 patients were included; 2 lacked progression data and 50 died before remission. Survival analysis was performed using Cox regression, Kaplan–Meier estimates, and log‐rank tests, with age, cytogenetic risk, and white blood cell (WBC) count as covariates. Only variables that were statistically significant in the univariate analysis were included in the multivariate model. A stepwise selection process was used for multivariate analysis. A bootstrap resampling tool was used for internal data validation [26]. Data analysis was performed using GraphPad Prism 10.0 and IBM SPSS Statistics 31, with *p* < 0.05 considered significant.

Cytogenetic risk and WBC count predicted OS and DFS, whereas age impacted only OS. Univariate analysis indicated that *RHOBTB2*, *RND3*, and *RHOF* influenced survival. Multivariate analysis demonstrated that higher *RHOBTB2* expression was an independent prognostic factor for worse OS (HR = 1.19, CI = 1.03–1.38; *p* < 0.05) and DFS (HR = 1.23, CI = 1.01–1.50; *p* < 0.05) (Table S3). Kaplan–Meier curves showed a 40% 5‐year OS for patients with lower *RHOBTB2* expression versus 17% for those with higher levels (*p* < 0.01) (Figure 2A). High *RHOBTB2* expression further related with shorter DFS (*p* < 0.05) (Figure 2B) and inferior OS in the intermediate‐risk cytogenetic group (*p* < 0.05) (Figure 2C). *RHOBTB2* expression did not differ among patients stratified according to cytogenetic risk (Figure 2D), whereas the expression of *RND1*, *RND3*, *RHOQ*, *CDC42*, and *RHOH* varied across these groups (Figure S1).

**FIGURE 2:**
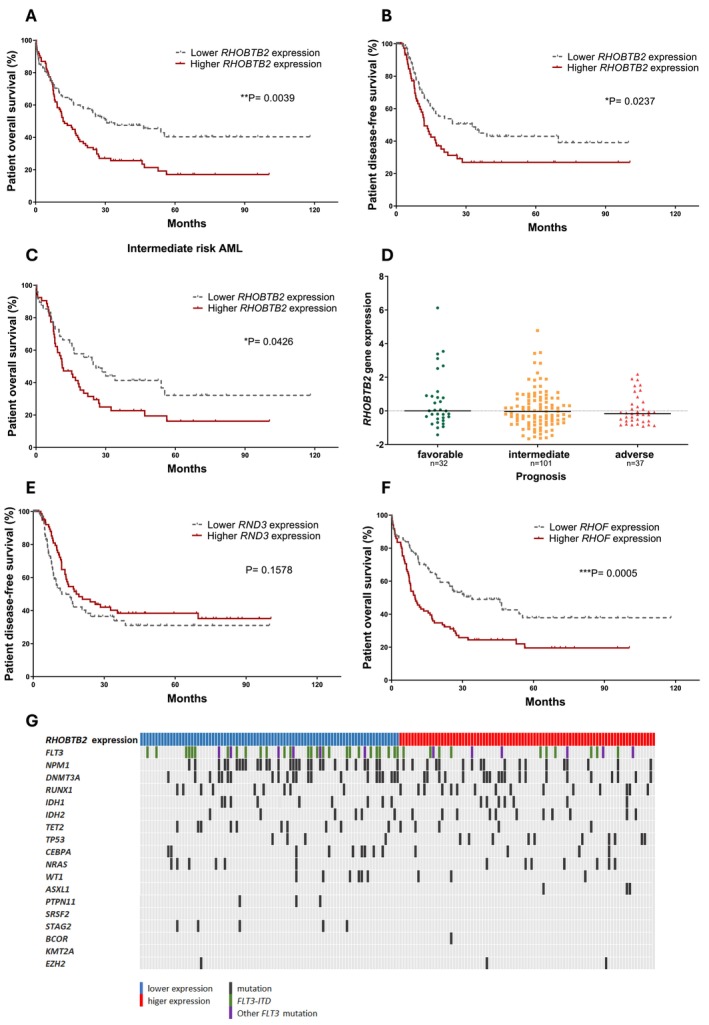
Impact of *RHOBTB2*, *RND3* and *RHOF* gene expression on AML patient survival. Data obtained from the TCGA study. (A‐C) Kaplan–Meier curves showing overall survival (OS) and disease‐free survival (DFS) in *de novo* AML patients stratified by *RHOBTB2*, (E) *RND3* and (F) *RHOF* expression. Patients were divided into high and low expression groups based on the median expression level. *p*‐values are shown (Log‐rank Mantel‐Cox test). (D) *RHOBTB2* expression in AML patients stratified by cytogenetic risk categories (*n* = 170, 3 cases lacked cytogenetic risk data). (G) Recurrent somatic mutations in individual AML patients, ordered by ascending *RHOBTB2* expression. Patients were grouped into high and low *RHOBTB2* expression categories using the median as cutoff. Each column represents one patient; mutations are indicated by black vertical lines. Patients with *FLT3‐ITD* mutations are shown in green, and those with *FLT3‐TKD* or mutations in other *FLT3* domains are shown in purple.

According to Kaplan–Meier analysis, *RND*3 expression did not impact survival (Figure 2E). Patients with higher *RHOF* showed worse prognosis, with median 5‐year OS of 19% versus 38% in those with lower expression (*p* < 0.001) (Figure 2F).

Since recurrent genetic mutations are important for AML risk stratification [27, 28], we evaluated associations of *RHOBTB2* expression with mutation profiles in the TCGA cohort. High *RHOBTB2* expression associated with a lower frequency of *FLT3‐ITD* mutations (Figure 2G, Table S4), while no associations were observed with other mutations.

RHOBTB2 (also known as DBC2) encodes an atypical RHO GTPase that interacts with Cullin‐3 and participates in proteasome‐mediated degradation [29]. It has been described as a tumor suppressor in solid tumors, being downregulated in breast, bladder, gastric, lung, and bone cancers [30, 31, 32, 33, 34]. RHOBTB2 tumor suppressive functions have been attributed to MSI2 ubiquitination [35] and cyclin D1 downregulation [36]. In contrast, our data, together with previous reports [37, 38], indicate that *RHOBTB2* is upregulated in AML. We additionally detected increased levels in MDS‐LB, reported here for the first time. Mechanistically, RHOBTB2 has been proposed as an E2F1 target gene, showing dual effects on proliferation depending on its expression dynamics [39]. We found that high *RHOBTB2* expression predicted inferior OS and DFS, including in the intermediate‐risk group. These findings are supported by mechanistic evidence demonstrating that RHOBTB2 overexpression promotes leukemic cell proliferation and migration, shortens the G0/G1 phase, and attenuates apoptosis via the RHOBTB2–KLHL13–YAP signaling axis [40]. Of note, *RHOBTB2* expression was also associated with a lower frequency of *FLT3‐ITD* mutations, which confer adverse prognosis [41]. Thus, the impact of RHOBTB2 on survival does not appear to be linked to recurrent mutations, supporting an independent oncogenic role in AML.

The RND subfamily members (RND1, RND2, RND3) share significant homology and induce cell rounding [42, 43]. RND3 is broadly expressed, whereas RND1 and RND2 are tissue restricted [44]. In our cohort, *RND1* was higher in MDS‐IB versus MDS‐LB, and *RND2* was elevated in *de novo* AML. *RND1* (in MDS) and *RND2* (in AML) showed moderate positive correlations with blast percentage, suggesting that blast burden may partially influence their expression in whole bone marrow samples.

*RND1* upregulation has also been reported in esophageal, breast, pancreatic, and lymphoid tumors [42, 43, 45], while reduced expression was related to hepatocellular carcinoma progression [46]. Similarly, *RND2* is increased in brain tumors and thymoma but downregulated in several epithelial cancers [43]. In glioblastoma, RND2 inhibits p38/MAPK signaling, impairing apoptosis and autophagy, thereby promoting tumor growth [47]. However, p38 inhibition in myeloid cell lines reduced cell growth, induced apoptosis, and enhanced chemosensitivity [48], pointing to an oncogenic p38 function. Despite their altered expression, neither *RND1* nor *RND2* presented prognostic value in AML, and whether they exert pro‐ or anti‐oncogenic roles in myeloid cells remains unresolved.

*RHOQ* (TC10) was downregulated in AML‐MRC and *de novo* AML compared with controls. Functionally, RHOQ is implicated in insulin signaling [49] and neurite outgrowth [50]. In cancer, it has been linked to metastasis in breast carcinoma [51] and Ras‐driven transformation [52], whereas reduced RHOQ expression enhanced invasion of lung adenocarcinoma cells through TGF‐β/Smad suppression [53]. Despite its reduced expression in AML, no association with patient survival was detected.

*RHOF* expression did not differ between patient groups. However, higher levels were associated with worse OS in the TCGA cohort. RHOF regulates cytoskeletal remodeling and vesicle trafficking [54] and has been proposed as a biomarker in lymphoma [55] and AML [54]. Its overexpression promoted proliferation and chemotherapy resistance in myeloid cells [56], reinforcing an oncogenic function.

In summary, we identified distinct patterns of gene expression among MDS and AML patients, highlighting *RHOBTB2*, *RND2*, and *RHOQ* as differentially expressed in disease. *RHOBTB2* stands out as a candidate prognostic marker associated with poor outcomes. Notably, RHOBTB and RND proteins are atypical GTPases regulated mainly at the transcriptional and protein turnover levels rather than by canonical GDP/GTP cycling, rendering their expression particularly informative of biological activity.

As commonly observed in other RHO GTPases, the roles of RHOBTB2, RND2, and RHOQ appear highly context dependent. This behavior highlights their functional complexity and underscores the importance of cellular context in determining biological impact. A comprehensive characterization of the proteins encoded by these genes in myeloid cells is beyond the scope of this study; nonetheless, our findings highlight their potential significance as biomarkers in AML.

## Author Contributions

B.A.R., M.C.C.R., and A.F.D. drafted the manuscript. L.B.P. and B.A.R. performed the experiments and conducted data analysis and interpretation. S.T.O.S. and M.L. critically revised the manuscript for important intellectual content and supervised the project. All authors contributed to the study design, reviewed and approved the final version of the manuscript, and agreed to be accountable for all aspects of the work.

## Funding

This work was supported by Fundação de Amparo à Pesquisa do Estado de São Paulo (Grants 2021/14630‐2 and 2022/03171‐0), Coordenação de Aperfeiçoamento de Pessoal de Nível Superior, Financial code 001.

## Ethics Statement

This study was approved by the local ethics committees under protocol numbers #5.672.921 and #6.065.515.

## Conflicts of Interest

The authors declare no conflicts of interest.

## Supporting information


**Figure S1:** cam471770‐sup‐0001‐Supinfo.docx. Expression of nine RHO GTPase genes in de novo AML patients stratified by cytogenetic risk. Gene expression data were obtained from The Cancer Genome Atlas (TCGA) study (*n* = 170, as cytogenetic risk information was unavailable for 3 patients). Each dot represents one patient, and horizontal lines indicate medians. The numbers of patients in each group and the *p* values (Mann–Whitney test) are indicated. (A) *RND1* gene expression was increased in the favorable‐risk group compared to the intermediate‐ and adverse‐risk groups. (C) *RND3* expression was decreased in the adverse‐risk group compared to the favorable‐ and intermediate‐risk groups. (D–E) *RHOQ* and *CDC42* expression were decreased in the favorable‐risk group compared to the adverse‐risk group. (F) *RHOH* gene expression was increased in the favorable‐risk group compared to the adverse‐risk group. (B, G–I) Expression of *RND2*, *RHOF*, *RHOU*, and *RHOV* did not significantly differ among the groups.
**Table S1:** Characteristics of Study Participants.
**Table S2:** Identification of probes used to evaluate RHO GTPase gene expression by the TaqMan system.
**Table S3:** Univariate and multivariate analysis for OS and DFS of AML patients from the TCGA cohort according to *RHO GTPase* expression.
**Table S4:** Association between *RHOBTB2* expression and recurrent mutations in AML.

## Data Availability

The datasets generated during the current study are available from the corresponding author upon reasonable request. The data that support the findings of this study are available in The Cancer Genome Atlas (TCGA) at https://www.cancer.gov/tcga.
