# Supplementary material for: Profiling Ten RHO GTPases in Myeloid Malignancies Reveals Distinct Expression Patterns and Prognostic Associations
Source: Cancer Med. 2026 Apr 2;15(4):e71770. doi: 10.1002/cam4.71770 (PMC13052033; doi:10.1002/cam4.71770)
Supplement: Supplementary file 1 — Figure S1: cam471770‐sup‐0001‐Supinfo.docx. Expression of nine RHO GTPase genes in de novo AML patients stratified by cytogenetic risk. Gene expression data were obtained from The Cancer Genome Atlas (TCGA) study (n = 170, as cytogenetic risk information was unavailable for 3 patients). Each dot represents one patient, and horizontal lines indicate medians. The numbers of patients in each group and the p values (Mann–Whitney test) are indicated. (A) RND1 gene expression was increased in the favorable‐risk group compared to the intermediate‐ and adverse‐risk groups. (C) RND3 expression was decreased in the adverse‐risk group compared to the favorable‐ and intermediate‐risk groups. (D–E) RHOQ and CDC42 expression were decreased in the favorable‐risk group compared to the adverse‐risk group. (F) RHOH gene expression was increased in the favorable‐risk group compared to the adverse‐risk group. (B, G–I) Expression of RND2, RHOF, RHOU, and RHOV did not significantly differ among the groups. Table S1: Characteristics of Study Participants. Table S2: Identification of probes used to evaluate RHO GTPase gene expression by the TaqMan system. Table S3: Univariate and multivariate analysis for OS and DFS of AML patients from the TCGA cohort according to RHO GTPase expression. Table S4: Association between RHOBTB2 expression and recurrent mutations in AML. [file CAM4-15-e71770-s001.docx]

**Profiling Ten RHO GTPases in Myeloid Malignancies Reveals Distinct Expression Patterns and Prognostic Associations**

**Running title:** *Expression of RHO GTPases in myeloid malignancies*

Beatriz de Almeida Rodrigues^2*^, Luciana Bueno de Paiva^1*^; Maria Carolina Clares Ramalho^2^, Amanda Ferreira Damasceno^2^, Sara Teresinha Olalla Saad^1^, Mariana Lazarini^2^.

*Beatriz de Almeida Rodrigues and Luciana Bueno de Paiva contributed equally to this manuscript.

(1) Hematology and Blood Transfusion Center, National Institute of Science and Technology of Blood (INCTS), University of Campinas (UNICAMP), Campinas, Brazil.

(2) Department of Clinical and Experimental Oncology, Federal University of São Paulo (UNIFESP), São Paulo, Brazil.

**Running title:** *Expression of Rho GTPases in Myeloid Malignancies*

This file contains:

Supplementary Figures

Supplementary Tables

# **
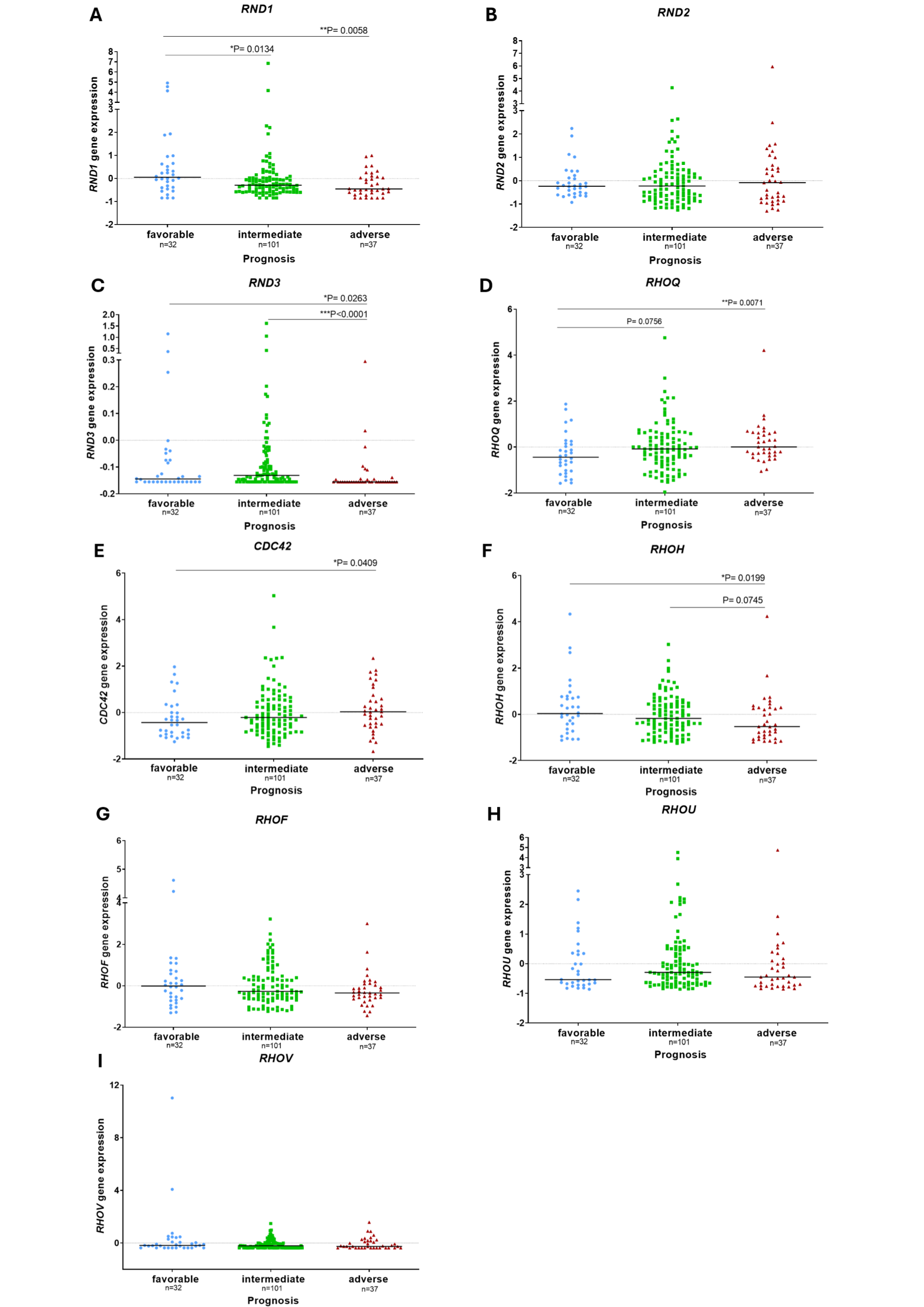
**

# **Supplementary Figure 1. Expression of nine RHO GTPase genes in de novo AML patients stratified by cytogenetic risk.** Gene expression data were obtained from The Cancer Genome Atlas (TCGA) study (n = 170, as cytogenetic risk information was unavailable for 3 patients). Each dot represents one patient, and horizontal lines indicate medians. The numbers of patients in each group and the P values (Mann–Whitney test) are indicated. (A) *RND1* gene expression was increased in the favorable-risk group compared to the intermediate- and adverse-risk groups. (C) *RND3* expression was decreased in the adverse-risk group compared to the favorable- and intermediate-risk groups. (D–E) *RHOQ* and *CDC42* expression were decreased in the favorable-risk group compared to the adverse-risk group. (F) *RHOH* gene expression was increased in the favorable-risk group compared to the adverse-risk group. (B, G–I) Expression of *RND2*, *RHOF*, *RHOU*, and *RHOV* did not significantly differ among the groups.

# **Supplementary Table 1.** Characteristics of Study Participants

| **Participants** | **Number** |  |  |
| --- | --- | --- | --- |
| **Healthy donors** | **14** |  |  |
| Sex |  |  |  |
| Male/Female | 9/5 |  |  |
| Age (years), median (range): | 31 (25-69) |  |  |
| **MDS** | **47** |  |  |
| Sex  Male/Female | 26/21 |  |  |
| Age (years), median (range): | 71 (20-86) |  |  |
| BM blast percentage, median (range) | 3 (0-20) |  |  |
| *WHO classification (morphologically defined):* |  |  |  |
| MDS-LB | 28 |  |  |
| MDS-IB1 | 12 |  |  |
| MDS-IB2 | 7 |  |  |
|  |  |  |  |
| *IPSS-R*^1^ |  |  |  |
| Low  Intermediate 1  Intermediate 2  High | 14  24  7  2 |  |  |
| Not available | 2 |  |  |
|  |  |  |  |
| **AML (UNICAMP cohort)** |  | **AML (TCGA cohort)** |  |
| *de novo* AML/AML-MR | 43/15 | 173 |  |
| Sex  Male/Female | 35/23 | 92/81 |  |
| Age (years), median (range): | 60 (22-90) | 58 (18-88) |  |
| BM blast percentage, median (range) | 56 (20-98) | 72 (30-100) |  |
| *Cytogenetic risk*^2^ |  |  |  |
| Favorable | 7 | 32 |  |
| Intermediate | 29 | 101 |  |
| Adverse | 13 | 37 |  |
| Not available | 9 | 3 |  |

Abbreviations: AML, acute myeloid leukemia; AML-MR: acute myeloid leukemia, myelodysplasia-related; MDS, myelodysplastic neoplasms; MDS-LB: myelodysplastic neoplasm with low blasts; MDS-IB: myelodysplastic neoplasm with increased blasts; BM: bone marrow; NA, non-available; WHO, World Health Organization; IPSS-R, Revised International Prognostic Scoring System.

^1^In MDS cohort, karyotype findings included very good: -Y (n=1), good: normal (n=52), del(5q) (n=2); intermediate: +8 (n=3), -7 (n=1), other (n=2); poor: three abnormalities (n=1); and very poor: >3 abnormalities (n=1).

^2^In AML cohort, risk karyotype included t(8;21) (n=4) and inv(16) (n=1), intermediate risk included normal (n=27), trisomy 8 (n=4) and other abnormalities (n=5), and high risk included complex karyotype (n=9), del(5q) (n=2) and -7 (n=2).

**Supplementary Table 2.** Identification of probes used to evaluate RHO GTPase gene expression by the TaqMan system.

| Gene | Catalog number |
| --- | --- |
| *RHOBTB2* | Hs01598083_m1 |
| *RND1* | Hs00205507_m1 |
| *RND2* | Hs00183269_m1 |
| *RND3* | Hs01003594_m1 |
| *RHOQ* | Hs00865365_s1 |
| *CDC42* | Hs00918044_g1 |
| *RHOH* | Hs01877256_s1 |
| *RHOF* | Hs00368032_m1 |
| *RHOU* | Hs00221873_m1 |
| *RHOV* | Hs00370444_g1 |
| *HPRT1* | Hs02800695_m1 |

**Supplementary Table 3.** Univariate and multivariate analysis for OS and DFS of AML patients from the TCGA cohort according to *RHO GTPase* expression.

|  | **Overall survival (n=173)** | | | | | | **Disease-free survival (n=121)** | | | | | |
| --- | --- | --- | --- | --- | --- | --- | --- | --- | --- | --- | --- | --- |
|  | **Univariate analysis** | | | **Multivariate analysis** | | | **Univariate analysis** | | | **Multivariate analysis** | | |
| **Factor** | **HR**^a^ | **95% C.I.** | ***P*** | **HR** | **95% C.I.** | ***P*** | **HR** | **95% C.I.** | ***P*** | **HR** | **95% C.I.** | ***P*** |
| **Diagnosis Age** | **1.040** | **1.025-1.055** | **<0.001** | **1.035** | **1.021-1.050** | **<0.001** | 1.014 | 0.998-1.029 | 0.081 |  |  |  |
| **Gender (M/F)** | 0.947 | 0.655-1.368 | 0.770 |  |  |  | 0.967 | 0.627-1.492 | 0.879 |  |  |  |
| **Cytogenetic risk**^b^ | **3.273** | **1.751-6.116** | **<0.001** | 1.911 | 0.993-3.678 | 0.052 | **3.305** | **1.699-6.429** | **<0.001** | **2.199** | **1.079-4.479** | **0.030** |
| **WBC count** | **1.005** | **1.001-1.008** | **0.018** | **1.006** | **1.002-1.011** | **0.003** | **1.006** | **1.002-1.011** | **0.007** | **1.006** | **1.001-1,010** | **0.019** |
| ***RHOBTB2*** | **1.330** | **1.162-1.523** | **<0.001** | **1.191** | **1.027-1.381** | **0.021** | **1.364** | **1.145-1.624** | **<0.001** | **1.231** | **1.008-1.503** | **0.042** |
| ***RND1*** | 1.076 | 0.914-1.268 | 0.378 |  |  |  | 1.028 | 0.835-1.264 | 0.797 |  |  |  |
| ***RND2*** | 0.845 | 0.686-1.041 | 0.113 |  |  |  | 0.944 | 0.761-1.170 | 0.598 |  |  |  |
| ***RND3*** | 1.116 | 0.962-1.294 | 0.147 |  |  |  | **1.189** | **1.025-1.380** | **0.022** | **1.191** | **1.023-1.387** | **0.024** |
| ***RHOQ*** | 0.986 | 0.821-1.184 | 0.879 |  |  |  | 1.085 | 0.900-1.309 | 0.393 |  |  |  |
| ***CDC42*** | 1.038 | 0.878-1.226 | 0.664 |  |  |  | 1.044 | 0.867-1.258 | 0.647 |  |  |  |
| ***RHOH*** | 1.051 | 0.870-1.270 | 0.607 |  |  |  | 1.036 | 0.829-1.296 | 0.754 |  |  |  |
| ***RHOF*** | **1.360** | **1.159-1.596** | **<0.001** | **1.193** | **1.011-1.408** | **0.037** | **1.360** | **1.107-1.671** | **0.003** | 1.212 | 0.962-1.528 | 0.103 |
| ***RHOU*** | 1.102 | 0.907-1.338 | 0.330 |  |  |  | 1.074 | 0.850-1.357 | 0.548 |  |  |  |
| ***RHOV*** | 1.138 | 0.948-1.365 | 0.165 |  |  |  | 0.851 | 0.513-1.413 | 0.534 |  |  |  |

Abbreviations: TCGA. The Cancer Genome Atlas. DFS. disease-free survival; OS. overall survival.

Statistically significant difference is highlighted in bold. Age, white blood cells (WBC) count and gene expression were analyzed for continuous variation.

^a^Hazard ratios (HR) > 1 indicate that increasing values for continuous variable or the first factor for categorical variable has the poorer outcome.

^b^Missing values were excluded in the calculation of p -values.

**Supplementary Table 4**. Association between *RHOBTB2* expression and recurrent mutations in AML.

| Gene mutated | Total of patients  n= 173 | Low *RHOBTB2* expression  n= 87 | High *RHOBTB2* expression  n= 86 | P-value^b^ |
| --- | --- | --- | --- | --- |
| *FLT3-ITD, n (%)* | 37 (21) | 27 (31) | 10 (12) | **P= 0.0027** |
| *FLT3-TKD, n (%)* | 12 (16) | 6 (7) | 6 (7) | P> 0.9999 |
| *npm1, n (%)* | 48 (28) | 30 (35) | 18 (21) | P= 0.0615 |
| *dnmt3a, n (%)* | 42 (24) | 22 (25) | 20 (23) | P= 0.8596 |
| *runx1, n (%)* | 24 (14) | 9 (10) | 15 (17) | P= 0.1940 |
| *idh1, n (%)* | 16 (9) | 9 (10) | 7 (8) | P= 0.7939 |
| *idh2, n (%)* | 17 (10) | 6 (7) | 11 (13) | P= 0.2124 |
| *tet2, n (%)* | 15 (9) | 10 (11) | 5 (6) | P= 0.2798 |
| *tp53, n (%)* | 14 (8) | 4 (5) | 10 (12) | P= 0.1025 |
| *cEbpa, n (%)* | 13 (8) | 9 (10) | 4 (5) | P= 0.2482 |
| *nras, n (%)* | 12 (7) | 5 (6) | 7 (8) | P= 0.5659 |
| *wt1, n (%)* | 10 (6) | 7 (8) | 3 (3) | P= 0.1933 |
| *asxl1, n (%)* | 3 (2) | 0 (0) | 3 (3) | P= 0.1207 |
| *PTPN11, n (%)* | 3 (2) | 3 (3) | 0 (0) | P= 0.2457 |
| *SRSF2, n (%)* | 0 (0) | 0 (0) | 0 (0) | P> 0.9999 |
| *STAG2, n (%)* | 5 (3) | 5 (6) | 0 (0) | P= 0.0589 |
| *BCOR, n (%)* | 1 (1) | 0 (0) | 1 (1) | P= 0.4971 |
| *KMT2A, n (%)* | 0 (0) | 0 (0) | 0 (0) | P> 0.9999 |
| *EZH2, n (%)* | 3 (2) | 1 (1) | 2 | P= 0.6206 |

Abbreviations: TCGA. The Cancer Genome Atlas; AML. acute myeloid leukemia. Gene expression values were dichotomized by median.

^a^The clinical and laboratorial data of TCGA AML cohort were obtained from cBioPortal for Cancer Genomics (http://www.cbioportal.org).

^b^Fisher’s exact test. The statistically significant P-value is highlighted in bold.
